# 3,4,5-Trihydr­oxy-*N*′-(1*H*-indol-3-yl­methyl­idene)benzohydrazide penta­hydrate

**DOI:** 10.1107/S1600536808039342

**Published:** 2008-11-29

**Authors:** Hamid Khaledi, Hapipah Mohd Ali, Seik Weng Ng

**Affiliations:** a Department of Chemistry, University of Malaya, 50603 Kuala Lumpur, Malaysia

## Abstract

The two aromatic parts of the title compound, C_16_H_13_N_3_O_4_·5H_2_O, are connected through a conjugated –CH=N–NH–C(O)– fragment, giving an almost planar mol­ecule. The organic mol­ecules and uncoordinated water mol­ecules are linked by N—H⋯O and O—H⋯O hydrogen bonds into a three-dimensional network.

## Related literature

For the structure of anhydrous *N*′-(1*H*-indol-3-ylmethyl­idene)-3,4,5-trihydroxy­benzohydrazide, see: Khaledi *et al.* (2008 ▶).
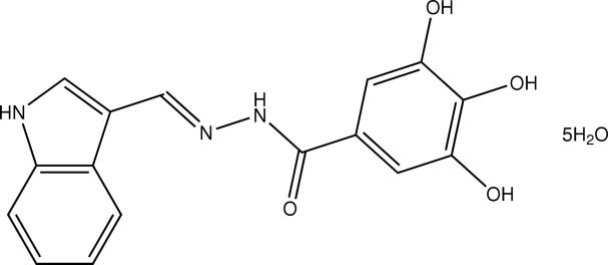


## Experimental

### 

#### Crystal data

C_16_H_13_N_3_O_4_·5H_2_O
*M* *_r_* = 401.37
Triclinic, 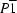

*a* = 7.4379 (2) Å
*b* = 9.1178 (2) Å
*c* = 14.1966 (3) Å
α = 103.814 (1)°
β = 103.716 (1)°
γ = 90.613 (2)°
*V* = 905.95 (4) Å^3^
*Z* = 2
Mo *K*α radiation
μ = 0.12 mm^−1^
*T* = 100 (2) K
0.30 × 0.25 × 0.04 mm

#### Data collection

Bruker SMART APEX diffractometer
Absorption correction: none
7524 measured reflections
4096 independent reflections
3285 reflections with *I* > 2σ(*I*)
*R*_int_ = 0.023

#### Refinement

*R*[*F* ^2^ > 2σ(*F* ^2^)] = 0.041
*wR*(*F* ^2^) = 0.115
*S* = 1.05
4096 reflections
313 parameters
15 restraints
H atoms treated by a mixture of independent and constrained refinement
Δρ_max_ = 0.35 e Å^−3^
Δρ_min_ = −0.25 e Å^−3^

### 

Data collection: *APEX2* (Bruker, 2007 ▶); cell refinement: *APEX2*; data reduction: *SAINT* (Bruker, 2007 ▶); program(s) used to solve structure: *SHELXS97* (Sheldrick, 2008 ▶); program(s) used to refine structure: *SHELXL97* (Sheldrick, 2008 ▶); molecular graphics: *X-SEED* (Barbour, 2001 ▶); software used to prepare material for publication: *pubCIF* (Westrip, 2008 ▶).

## Supplementary Material

Crystal structure: contains datablocks global, I. DOI: 10.1107/S1600536808039342/tk2335sup1.cif

Structure factors: contains datablocks I. DOI: 10.1107/S1600536808039342/tk2335Isup2.hkl

Additional supplementary materials:  crystallographic information; 3D view; checkCIF report

## Figures and Tables

**Table 1:** Hydrogen-bond geometry (Å, °)

*D*—H⋯*A*	*D*—H	H⋯*A*	*D*⋯*A*	*D*—H⋯*A*
O1—H1*o*⋯O4^i^	0.85 (1)	1.90 (1)	2.740 (2)	174 (2)
O2—H2*o*⋯O2*w*	0.84 (1)	1.94 (1)	2.697 (2)	149 (2)
O3—H3*o*⋯O3*w*	0.85 (1)	1.90 (1)	2.724 (2)	164 (2)
N1—H1*n*⋯O1*w*^ii^	0.88 (1)	2.11 (1)	2.978 (2)	169 (2)
N3—H3*n*⋯O4*w*^iii^	0.88 (1)	2.15 (1)	3.024 (2)	171 (2)
O1*w*—H11⋯O1	0.85 (1)	1.93 (1)	2.771 (2)	170 (3)
O1*w*—H12⋯O3*w*^iv^	0.86 (1)	1.94 (1)	2.760 (2)	161 (3)
O2*w*—H21⋯O3^v^	0.84 (1)	2.29 (1)	3.123 (2)	172 (2)
O2*w*—H22⋯O5*w*	0.85 (1)	1.94 (2)	2.753 (2)	161 (3)
O3*w*—H31⋯O5*w*^vi^	0.85 (1)	1.95 (1)	2.796 (2)	172 (3)
O3*w*—H32⋯O3^vii^	0.85 (1)	2.06 (1)	2.890 (2)	165 (2)
O4*w*—H41⋯O1*w*	0.85 (1)	1.97 (1)	2.803 (2)	169 (2)
O4*w*—H42⋯O4^viii^	0.84 (1)	2.37 (2)	2.921 (2)	124 (2)
O4*w*—H42⋯N2^viii^	0.84 (1)	2.39 (1)	3.211 (2)	166 (2)
O5*w*—H51⋯O4*w*^ix^	0.85 (1)	1.97 (1)	2.798 (2)	167 (2)
O5*w*—H52⋯O2*w*^x^	0.85 (1)	1.97 (1)	2.810 (2)	168 (3)

Symmetry codes: (i) 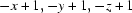; (ii) 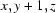; (iii) 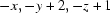; (iv) 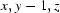; (v) 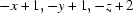; (vi) 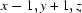; (vii) 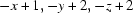; (viii) 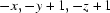; (ix) 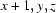; (x) 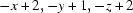.

**Fig. 1.**
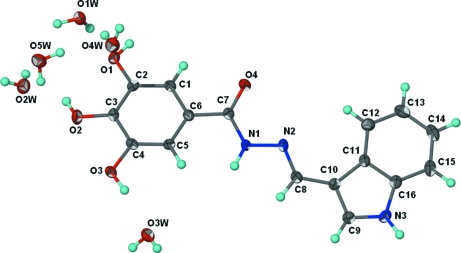
Thermal ellipsoid plot (Barbour, 2001) of C16H13N3O4.5H2O at the 70% probability level. Hydrogen atoms are drawn as spheres of arbitrary radius.

## supplementary crystallographic information

### Comment

(type here to add)

### Experimental

Indole-3-carbaldehyde (1.0 g, 7 mmol) and 3,4,5-trihydroxybenzoylhydrazine (1.29 g, 7 mmol) were heated in ethanol (60 ml) for 6 h. About 1 ml of acetic acid also added. The solution was set aside for the growth of crystals.

### Refinement

Hydrogen atoms were placed at calculated positions (C–H 0.95 Å), and were treated as riding on their parent carbon atoms, with *U*(H) set to 1.2*U*_eq_(C). The nitrogen- and oxygen-bound H atoms were located in a difference Fourier map, and were refined with distance restraints of N–H 0.88±0.01 and O–H 0.84±0.01 Å.

### Crystal data

**Table:**

C_16_H_13_N_3_O_4_·5H_2_O	*Z* = 2
*M**_r_* = 401.37	*F*_000_ = 424
Triclinic, *P*1	*D*_x_ = 1.471 Mg m^−^^3^
Hall symbol: -P 1	Mo *K*α radiation λ = 0.71073 Å
*a* = 7.4379 (2) Å	Cell parameters from 2461 reflections
*b* = 9.1178 (2) Å	θ = 2.3–28.0º
*c* = 14.1966 (3) Å	µ = 0.12 mm^−^^1^
α = 103.814 (1)º	*T* = 100 (2) K
β = 103.716 (1)º	Plate, pale-yellow
γ = 90.613 (2)º	0.30 × 0.25 × 0.04 mm
*V* = 905.95 (4) Å^3^	

### Data collection

**Table:**

Bruker SMART APEX diffractometer	3285 reflections with *I* > 2σ(*I*)
Radiation source: fine-focus sealed tube	*R*_int_ = 0.023
Monochromator: graphite	θ_max_ = 27.5º
*T* = 100(2) K	θ_min_ = 2.3º
ω scans	*h* = −9→9
Absorption correction: None	*k* = −11→11
7524 measured reflections	*l* = −18→18
4096 independent reflections	

### Refinement

**Table:**

Refinement on *F*^2^	Secondary atom site location: difference Fourier map
Least-squares matrix: full	Hydrogen site location: inferred from neighbouring sites
*R*[*F*^2^ > 2σ(*F*^2^)] = 0.041	H atoms treated by a mixture of independent and constrained refinement
*wR*(*F*^2^) = 0.115	*w* = 1/[σ^2^(*F*_o_^2^) + (0.0613*P*)^2^ + 0.1789*P*] where *P* = (*F*_o_^2^ + 2*F*_c_^2^)/3
*S* = 1.05	(Δ/σ)_max_ = 0.001
4096 reflections	Δρ_max_ = 0.35 e Å^−^^3^
313 parameters	Δρ_min_ = −0.25 e Å^−^^3^
15 restraints	Extinction correction: none
Primary atom site location: structure-invariant direct methods	

### Fractional atomic coordinates and isotropic or equivalent isotropic displacement parameters (Å^2^)

**Table:**

	*x*	*y*	*z*	*U*_iso_*/*U*_eq_	
O1	0.59471 (16)	0.40029 (12)	0.72041 (8)	0.0184 (2)	
O2	0.51512 (17)	0.56940 (13)	0.89648 (8)	0.0217 (3)	
O3	0.35915 (16)	0.82334 (12)	0.90287 (8)	0.0180 (2)	
O4	0.31349 (16)	0.68239 (12)	0.45992 (8)	0.0197 (3)	
O1w	0.35758 (17)	0.17412 (13)	0.73409 (8)	0.0200 (3)	
O2w	0.75855 (17)	0.38643 (13)	0.97416 (9)	0.0221 (3)	
O3w	0.34432 (17)	1.12598 (13)	0.91713 (8)	0.0213 (3)	
O4w	0.01511 (18)	0.30256 (13)	0.69710 (9)	0.0231 (3)	
O5w	1.07168 (17)	0.33392 (13)	0.90316 (9)	0.0232 (3)	
N1	0.29295 (19)	0.91884 (14)	0.55157 (9)	0.0162 (3)	
N2	0.24067 (18)	0.97264 (14)	0.46592 (9)	0.0165 (3)	
N3	0.08352 (19)	1.39294 (15)	0.34212 (10)	0.0183 (3)	
C1	0.4530 (2)	0.58425 (16)	0.63391 (11)	0.0147 (3)	
H1	0.4753	0.5274	0.5729	0.018*	
C2	0.5040 (2)	0.53102 (16)	0.71918 (11)	0.0146 (3)	
C3	0.4698 (2)	0.61229 (17)	0.80875 (11)	0.0151 (3)	
C4	0.3855 (2)	0.74827 (16)	0.81103 (11)	0.0147 (3)	
C5	0.3332 (2)	0.80233 (16)	0.72644 (11)	0.0148 (3)	
H5	0.2733	0.8942	0.7290	0.018*	
C6	0.3693 (2)	0.72070 (16)	0.63685 (10)	0.0140 (3)	
C7	0.3236 (2)	0.77172 (16)	0.54225 (11)	0.0144 (3)	
C8	0.2179 (2)	1.11562 (17)	0.48486 (11)	0.0168 (3)	
H8	0.2354	1.1700	0.5528	0.020*	
C9	0.1240 (2)	1.34618 (17)	0.42795 (11)	0.0175 (3)	
H9	0.1226	1.4073	0.4921	0.021*	
C10	0.1675 (2)	1.19684 (17)	0.40875 (11)	0.0158 (3)	
C11	0.1553 (2)	1.14989 (17)	0.30295 (11)	0.0154 (3)	
C12	0.1934 (2)	1.01736 (17)	0.23897 (11)	0.0194 (3)	
H12A	0.2299	0.9310	0.2632	0.023*	
C13	0.1763 (2)	1.01605 (19)	0.13982 (12)	0.0229 (4)	
H13	0.2023	0.9275	0.0956	0.028*	
C14	0.1213 (2)	1.1422 (2)	0.10270 (12)	0.0241 (4)	
H14	0.1093	1.1367	0.0338	0.029*	
C15	0.0845 (2)	1.27369 (19)	0.16400 (12)	0.0212 (3)	
H15	0.0478	1.3594	0.1390	0.025*	
C16	0.1033 (2)	1.27600 (17)	0.26438 (11)	0.0169 (3)	
H1o	0.616 (3)	0.370 (2)	0.6634 (10)	0.046 (7)*	
H2o	0.584 (3)	0.4971 (18)	0.8974 (16)	0.039 (6)*	
H3o	0.333 (3)	0.9129 (14)	0.8998 (16)	0.038 (6)*	
H1n	0.314 (3)	0.9850 (17)	0.6102 (9)	0.025 (5)*	
H3n	0.052 (3)	1.4844 (15)	0.3374 (17)	0.046 (6)*	
H11	0.438 (3)	0.235 (2)	0.7274 (19)	0.060 (8)*	
H12	0.380 (4)	0.163 (3)	0.7939 (10)	0.059 (8)*	
H21	0.719 (3)	0.336 (2)	1.0090 (16)	0.055 (7)*	
H22	0.848 (3)	0.349 (4)	0.952 (2)	0.112 (13)*	
H31	0.255 (3)	1.184 (3)	0.915 (2)	0.067 (9)*	
H32	0.425 (2)	1.158 (2)	0.9721 (10)	0.037 (6)*	
H41	0.117 (2)	0.260 (3)	0.7001 (18)	0.055 (8)*	
H42	−0.066 (3)	0.242 (2)	0.6537 (15)	0.063 (8)*	
H51	1.039 (3)	0.331 (3)	0.8414 (8)	0.055 (8)*	
H52	1.124 (4)	0.4219 (19)	0.932 (2)	0.101 (12)*	

### Atomic displacement parameters (Å^2^)

**Table:**

	*U*^11^	*U*^22^	*U*^33^	*U*^12^	*U*^13^	*U*^23^
O1	0.0248 (6)	0.0168 (5)	0.0172 (6)	0.0079 (5)	0.0091 (5)	0.0068 (4)
O2	0.0281 (7)	0.0247 (6)	0.0164 (6)	0.0119 (5)	0.0081 (5)	0.0099 (5)
O3	0.0241 (6)	0.0179 (6)	0.0123 (5)	0.0060 (5)	0.0060 (4)	0.0026 (4)
O4	0.0282 (6)	0.0169 (5)	0.0131 (5)	0.0037 (5)	0.0051 (5)	0.0021 (4)
O1w	0.0247 (6)	0.0202 (6)	0.0156 (6)	0.0008 (5)	0.0053 (5)	0.0051 (4)
O2w	0.0242 (7)	0.0240 (6)	0.0223 (6)	0.0054 (5)	0.0101 (5)	0.0094 (5)
O3w	0.0235 (6)	0.0233 (6)	0.0155 (6)	0.0042 (5)	0.0031 (5)	0.0032 (4)
O4w	0.0217 (6)	0.0249 (6)	0.0217 (6)	0.0056 (5)	0.0035 (5)	0.0057 (5)
O5w	0.0249 (7)	0.0234 (6)	0.0203 (6)	0.0028 (5)	0.0070 (5)	0.0023 (5)
N1	0.0228 (7)	0.0147 (6)	0.0106 (6)	0.0027 (5)	0.0029 (5)	0.0037 (5)
N2	0.0201 (7)	0.0179 (6)	0.0127 (6)	0.0027 (5)	0.0041 (5)	0.0063 (5)
N3	0.0196 (7)	0.0150 (6)	0.0212 (7)	0.0033 (5)	0.0041 (5)	0.0069 (5)
C1	0.0158 (7)	0.0148 (7)	0.0129 (7)	0.0006 (6)	0.0040 (6)	0.0019 (5)
C2	0.0143 (7)	0.0127 (7)	0.0176 (7)	0.0014 (6)	0.0046 (6)	0.0043 (6)
C3	0.0144 (7)	0.0181 (7)	0.0138 (7)	0.0005 (6)	0.0034 (6)	0.0060 (6)
C4	0.0140 (7)	0.0166 (7)	0.0126 (7)	−0.0003 (6)	0.0046 (6)	0.0009 (5)
C5	0.0158 (7)	0.0131 (7)	0.0152 (7)	0.0019 (6)	0.0040 (6)	0.0028 (5)
C6	0.0136 (7)	0.0148 (7)	0.0125 (7)	−0.0014 (6)	0.0019 (5)	0.0030 (5)
C7	0.0142 (7)	0.0159 (7)	0.0132 (7)	0.0002 (6)	0.0033 (6)	0.0037 (5)
C8	0.0191 (8)	0.0175 (7)	0.0136 (7)	0.0022 (6)	0.0041 (6)	0.0032 (6)
C9	0.0177 (8)	0.0176 (7)	0.0169 (7)	0.0013 (6)	0.0038 (6)	0.0043 (6)
C10	0.0164 (7)	0.0154 (7)	0.0155 (7)	0.0012 (6)	0.0035 (6)	0.0042 (6)
C11	0.0153 (7)	0.0175 (7)	0.0134 (7)	−0.0011 (6)	0.0027 (6)	0.0046 (6)
C12	0.0202 (8)	0.0174 (7)	0.0194 (8)	−0.0010 (6)	0.0042 (6)	0.0031 (6)
C13	0.0249 (9)	0.0226 (8)	0.0191 (8)	−0.0037 (7)	0.0070 (7)	−0.0005 (6)
C14	0.0242 (9)	0.0322 (9)	0.0145 (7)	−0.0084 (7)	0.0028 (6)	0.0054 (6)
C15	0.0185 (8)	0.0264 (8)	0.0191 (8)	−0.0041 (7)	0.0002 (6)	0.0109 (6)
C16	0.0137 (7)	0.0184 (7)	0.0178 (7)	−0.0017 (6)	0.0016 (6)	0.0054 (6)

### Geometric parameters (Å, °)

**Table:**

O1—C2	1.3777 (18)	C1—C2	1.383 (2)
O1—H1o	0.845 (9)	C1—C6	1.393 (2)
O2—C3	1.3615 (18)	C1—H1	0.9500
O2—H2o	0.839 (10)	C2—C3	1.392 (2)
O3—C4	1.3801 (17)	C3—C4	1.392 (2)
O3—H3o	0.851 (10)	C4—C5	1.379 (2)
O4—C7	1.2400 (17)	C5—C6	1.399 (2)
O1w—H11	0.850 (10)	C5—H5	0.9500
O1w—H12	0.855 (10)	C6—C7	1.490 (2)
O2w—H21	0.843 (10)	C8—C10	1.433 (2)
O2w—H22	0.846 (10)	C8—H8	0.9500
O3w—H31	0.854 (10)	C9—C10	1.381 (2)
O3w—H32	0.848 (10)	C9—H9	0.9500
O4w—H41	0.848 (10)	C10—C11	1.441 (2)
O4w—H42	0.839 (10)	C11—C12	1.404 (2)
O5w—H51	0.847 (10)	C11—C16	1.408 (2)
O5w—H52	0.850 (10)	C12—C13	1.380 (2)
N1—C7	1.3434 (19)	C12—H12A	0.9500
N1—N2	1.3910 (17)	C13—C14	1.402 (2)
N1—H1n	0.882 (9)	C13—H13	0.9500
N2—C8	1.288 (2)	C14—C15	1.375 (2)
N3—C9	1.354 (2)	C14—H14	0.9500
N3—C16	1.376 (2)	C15—C16	1.394 (2)
N3—H3n	0.882 (10)	C15—H15	0.9500
			
C2—O1—H1o	106.5 (16)	C5—C6—C7	123.54 (13)
C3—O2—H2o	115.8 (15)	O4—C7—N1	122.36 (13)
C4—O3—H3o	107.5 (14)	O4—C7—C6	121.46 (13)
H11—O1w—H12	112 (2)	N1—C7—C6	116.18 (13)
H21—O2w—H22	114 (3)	N2—C8—C10	123.34 (14)
H31—O3w—H32	109 (2)	N2—C8—H8	118.3
H41—O4w—H42	107 (2)	C10—C8—H8	118.3
H51—O5w—H52	106 (3)	N3—C9—C10	110.20 (14)
C7—N1—N2	119.24 (12)	N3—C9—H9	124.9
C7—N1—H1n	122.5 (12)	C10—C9—H9	124.9
N2—N1—H1n	118.0 (12)	C9—C10—C8	123.34 (14)
C8—N2—N1	113.26 (12)	C9—C10—C11	106.25 (13)
C9—N3—C16	109.03 (13)	C8—C10—C11	130.40 (14)
C9—N3—H3n	125.2 (15)	C12—C11—C16	119.25 (14)
C16—N3—H3n	125.7 (15)	C12—C11—C10	134.26 (14)
C2—C1—C6	120.49 (13)	C16—C11—C10	106.36 (13)
C2—C1—H1	119.8	C13—C12—C11	118.14 (15)
C6—C1—H1	119.8	C13—C12—H12A	120.9
O1—C2—C1	122.20 (13)	C11—C12—H12A	120.9
O1—C2—C3	117.48 (13)	C12—C13—C14	121.62 (15)
C1—C2—C3	120.29 (14)	C12—C13—H13	119.2
O2—C3—C4	116.52 (13)	C14—C13—H13	119.2
O2—C3—C2	124.59 (14)	C15—C14—C13	121.35 (15)
C4—C3—C2	118.89 (13)	C15—C14—H14	119.3
O3—C4—C5	123.17 (14)	C13—C14—H14	119.3
O3—C4—C3	115.42 (13)	C14—C15—C16	117.23 (15)
C5—C4—C3	121.41 (13)	C14—C15—H15	121.4
C4—C5—C6	119.40 (14)	C16—C15—H15	121.4
C4—C5—H5	120.3	N3—C16—C15	129.42 (14)
C6—C5—H5	120.3	N3—C16—C11	108.14 (13)
C1—C6—C5	119.50 (13)	C15—C16—C11	122.40 (15)
C1—C6—C7	116.96 (13)		
			
C7—N1—N2—C8	−179.15 (14)	N1—N2—C8—C10	178.78 (14)
C6—C1—C2—O1	−176.94 (13)	C16—N3—C9—C10	−1.24 (18)
C6—C1—C2—C3	0.9 (2)	N3—C9—C10—C8	179.52 (14)
O1—C2—C3—O2	−1.9 (2)	N3—C9—C10—C11	0.96 (18)
C1—C2—C3—O2	−179.82 (14)	N2—C8—C10—C9	172.92 (15)
O1—C2—C3—C4	177.27 (13)	N2—C8—C10—C11	−8.9 (3)
C1—C2—C3—C4	−0.7 (2)	C9—C10—C11—C12	175.33 (17)
O2—C3—C4—O3	0.3 (2)	C8—C10—C11—C12	−3.1 (3)
C2—C3—C4—O3	−178.94 (13)	C9—C10—C11—C16	−0.34 (17)
O2—C3—C4—C5	−179.82 (13)	C8—C10—C11—C16	−178.76 (16)
C2—C3—C4—C5	1.0 (2)	C16—C11—C12—C13	−0.6 (2)
O3—C4—C5—C6	178.43 (13)	C10—C11—C12—C13	−175.81 (16)
C3—C4—C5—C6	−1.5 (2)	C11—C12—C13—C14	−0.4 (2)
C2—C1—C6—C5	−1.4 (2)	C12—C13—C14—C15	0.9 (3)
C2—C1—C6—C7	178.38 (13)	C13—C14—C15—C16	−0.3 (2)
C4—C5—C6—C1	1.7 (2)	C9—N3—C16—C15	−176.86 (16)
C4—C5—C6—C7	−178.10 (13)	C9—N3—C16—C11	0.99 (17)
N2—N1—C7—O4	2.7 (2)	C14—C15—C16—N3	176.83 (16)
N2—N1—C7—C6	−176.87 (12)	C14—C15—C16—C11	−0.8 (2)
C1—C6—C7—O4	18.6 (2)	C12—C11—C16—N3	−176.83 (14)
C5—C6—C7—O4	−161.64 (14)	C10—C11—C16—N3	−0.38 (17)
C1—C6—C7—N1	−161.86 (13)	C12—C11—C16—C15	1.2 (2)
C5—C6—C7—N1	17.9 (2)	C10—C11—C16—C15	177.65 (14)

### Hydrogen-bond geometry (Å, °)

**Table:**

*D*—H···*A*	*D*—H	H···*A*	*D*···*A*	*D*—H···*A*
O1—H1o···O4^i^	0.85 (1)	1.90 (1)	2.740 (2)	174 (2)
O2—H2o···O2w	0.84 (1)	1.94 (1)	2.697 (2)	149 (2)
O3—H3o···O3w	0.85 (1)	1.90 (1)	2.724 (2)	164 (2)
N1—H1n···O1w^ii^	0.88 (1)	2.11 (1)	2.978 (2)	169 (2)
N3—H3n···O4w^iii^	0.88 (1)	2.15 (1)	3.024 (2)	171 (2)
O1w—H11···O1	0.85 (1)	1.93 (1)	2.771 (2)	170 (3)
O1w—H12···O3w^iv^	0.86 (1)	1.94 (1)	2.760 (2)	161 (3)
O2w—H21···O3^v^	0.84 (1)	2.29 (1)	3.123 (2)	172 (2)
O2w—H22···O5w	0.85 (1)	1.94 (2)	2.753 (2)	161 (3)
O3w—H31···O5w^vi^	0.85 (1)	1.95 (1)	2.796 (2)	172 (3)
O3w—H32···O3^vii^	0.85 (1)	2.06 (1)	2.890 (2)	165 (2)
O4w—H41···O1w	0.85 (1)	1.97 (1)	2.803 (2)	169 (2)
O4w—H42···O4^viii^	0.84 (1)	2.37 (2)	2.921 (2)	124 (2)
O4w—H42···N2^viii^	0.84 (1)	2.39 (1)	3.211 (2)	166 (2)
O5w—H51···O4w^ix^	0.85 (1)	1.97 (1)	2.798 (2)	167 (2)
O5w—H52···O2w^x^	0.85 (1)	1.97 (1)	2.810 (2)	168 (3)

Symmetry codes: (i) −*x*+1, −*y*+1, −*z*+1; (ii) *x*, *y*+1, *z*; (iii) −*x*, −*y*+2, −*z*+1; (iv) *x*, *y*−1, *z*; (v) −*x*+1, −*y*+1, −*z*+2; (vi) *x*−1, *y*+1, *z*; (vii) −*x*+1, −*y*+2, −*z*+2; (viii) −*x*, −*y*+1, −*z*+1; (ix) *x*+1, *y*, *z*; (x) −*x*+2, −*y*+1, −*z*+2.
